# The Omission of Nursing Care in Emergency Departments: A Conceptual Analysis Using Walker & Avant's Methodology

**DOI:** 10.1111/jan.17017

**Published:** 2025-05-01

**Authors:** Josiane Provost, Émilie Gosselin, Christian M. Rochefort

**Affiliations:** ^1^ Health Sciences Research Program Faculty of Medicine and Health Sciences, Université de Sherbrooke Sherbrooke Canada; ^2^ Nursing Université de Sherbrooke Sherbrooke Canada; ^3^ Faculty of Medicine and Health Sciences Université de Sherbrooke Sherbrooke Canada

**Keywords:** care rationing, concept analysis, emergency nursing, health policy, missed nursing care, nursing management, nursing shortages, patient safety, quality of care, workload

## Abstract

**Aim(s):**

To analyse the dimensions of the omission of nursing care in emergency departments, including its attributes, antecedents, and consequences, using Walker & Avant's concept analysis method.

**Design Concept Analysis:**

Methods: Walker and Avant's eight‐step method defined attributes, antecedents, and consequences of the omission of nursing care in emergency departments.

**Data Sources:**

A comprehensive literature review was conducted using CINAHL, MEDLINE, Embase, Health Management Database, and Cochrane Library, covering publications from 2001 to 2024. The search was conducted in August 2024.

**Results:**

Key attributes were delayed, incomplete, or interrupted care, mostly due to insufficient staffing or unpredictable patient volumes. Antecedents included high workloads, inadequate skill mixes, and understaffing. Consequences were increased patient morbidity and mortality, nurse burnout, and job dissatisfaction. A research gap exists in paediatric‐specific measurement tools.

**Conclusion:**

Identifying dimensions of omitted nursing care in emergency departments informs interventions to improve patient safety and care quality. Developing paediatric‐specific measurement tools is essential.

**Implications for the Profession and/or Patient Care:**

The findings emphasise the need for improved staffing and resource allocation policies, reducing risks to patients and enhancing nurse satisfaction.

**Impact:**

This study addressed the gap in understanding omitted nursing care specifically in emergency departments. Findings highlight systemic issues impacting patient outcomes and nurse well‐being. The results will guide organisational improvements and future research globally.

**Reporting Method:**

This study adhered to EQUATOR guidelines, following Walker and Avant's method for concept analysis.

**Patient or Public Contribution:**

This study did not include patient or public involvement.

**Impact Statement:**

This study underscores the critical impact of the omission of nursing care (ONC) in emergency departments (EDs) on patient safety, nurse well‐being, and healthcare efficiency. ONC contributes to increased morbidity, mortality, and adverse events, highlighting the urgent need for improved staffing models and resource allocation. Training programmes should equip emergency nurses with prioritisation strategies to mitigate care omissions. Policymakers must recognise ONC as a key quality indicator, ensuring adequate workforce support. Additionally, this study identifies a gap in measuring ONC in paediatric EDs, calling for the development of tailored assessment tools and further research on intervention strategies.

**Summary**

- What does this paper contribute to the wider global clinical community?
  ○ Highlights systemic factors causing care omission in emergency settings
  ○ Identifies a need for paediatric‐specific measurement tools.
  ○ Offers insights for improved staffing policies and patient safety.
- Why is this research or review needed?
  ○ Omission of nursing care has been extensively studied in acute care settings, but its specific manifestation and implications in emergency departments are less well understood.
  ○ The high‐stakes, time‐pressured nature of emergency care presents unique challenges that may increase the occurrence of omission of nursing care.
  ○ This analysis seeks to fill the gap in understanding how the omission of nursing care affects patient outcomes and nurse satisfaction in emergency departments.
- What are the key findings?
  ○ Omission of nursing care in emergency departments is influenced by systemic factors like understaffing, high patient volumes, and resource constraints, leading to incomplete or delayed nursing interventions.
  ○ There is an absence of tools specifically adapted to measure omission of nursing care in paediatric emergency care, which represents a critical gap in current research.
  ○ Omission of nursing care has significant implications for patient morbidity and nurse well‐being, necessitating organisational changes to mitigate its impact.
- How should the findings be used to influence policy/practice/research/education?
  ○ Healthcare organisations should implement staffing policies that account for the unique demands of emergency departments, particularly during peak times and in high‐stakes cases such as paediatric emergencies.
  ○ Training and education for emergency nurses should focus on prioritisation skills and strategies to manage high workloads and prevent care omissions.
  ○ Future research should develop validated tools that can specifically measure omission of nursing care in emergency contexts.

## 1 Introduction

The omission of nursing care (ONC), defined as ‘the complete or partial failure to perform necessary nursing tasks according to the patient's health status due to a lack of time, staffing, or appropriate skills’ (Kalisch et al. 2009; Schubert et al. 2008), is a major concern in healthcare settings globally (Aiken et al. 2018; Chaboyer et al. 2021). Internationally, ONC has emerged as a critical issue affecting patient safety, care quality, and health system efficiency, as evidenced by studies conducted in diverse countries and healthcare contexts. Indeed, its prevalence ranges from 55% to 98% depending on contexts and patient populations, significantly impacting patient safety and quality of care (Griffiths et al. 2018; Jones et al. 2015). Numerous international studies have shown a correlation between higher ONC rates and several negative patient outcomes, including increased morbidity, mortality, and adverse events (e.g., medication errors, falls, pressure ulcers, infections) (Aiken et al. 2018; Chaboyer et al. 2021; Recio‐Saucedo et al. 2018; Stemmer et al. 2022). ONC also has significant financial implications, leading to higher costs due to longer hospital stays and patient readmissions (Jones et al. 2015; Sasso et al. 2017).

Emergency departments (EDs) in particular are vulnerable to ONC due to the high‐pressure environment and the critical nature of the care provided (Fleet et al. 2019). The shortage of healthcare personnel, a widespread issue in EDs, increases the workload and stress on nursing staff, thereby elevating the risk of ONC (Fleet et al. 2019; Recio‐Saucedo et al. 2018). While research in acute hospital contexts has expanded to include medical and surgical wards and critical care, EDs are often not included (Duhalde et al. 2023). Common ONC cases in EDs include delays in administering pain medication, failure to provide patient education, omission of emotional support such as comforting anxious patients or family members, and incomplete discharge instructions (Duhalde et al. 2023). Comparing ONC in EDs with other settings (e.g., acute care, long‐term care) highlights distinct challenges. Unlike inpatient units, where care planning is structured over a shift, ED nurses must frequently reprioritise based on rapidly changing patient acuity. This unpredictability significantly impacts the type and frequency of ONC in EDs (Fleet et al. 2019).

Given these challenges, it is crucial to analyse the concept of ONC in EDs. Understanding the dimensions of ONC, including its attributes, antecedents, and consequences, can help healthcare professionals develop targeted interventions to mitigate its impact (Papathanasiou et al. 2024). By identifying the key factors contributing to ONC, particularly in high‐stakes environments like EDs, we can enhance patient safety, improve care quality, and optimise resource allocation. Moreover, the last conceptual analysis of ONC dates back 15 years (Kalisch et al. 2009). In recent years, we have witnessed significant changes in health policies, funding models, and quality of care requirements (World Health Organization 2021). This evolution may have impacted how ONC is perceived, measured, and managed in current healthcare settings (Ausserhofer et al. 2014). A contemporary analysis would highlight current trends and challenges faced by nurses (Walker and Avant 2005; Wilson 2010). Finally, re‐examining the concept of ONC can contribute to advancing knowledge and enriching existing literature (Rogers 1989; Walker and Avant 2005).

This work aims to analyse the dimensions of the omission of nursing care in emergency departments, including its attributes, antecedents and consequences, using Walker and Avant's concept analysis method. This analysis is based on an in‐depth review of existing literature and the use of proven methodological tools to evaluate and measure the omission of nursing care in the ED context.

## 2 Method

### 2.1 Study Design

This concept analysis was conducted using Walker and Avant's eight‐step method to analyse the meaning, attributes, dimensions, antecedents, and consequences of ONC. Concept analysis, while distinguishing a concept from other similar concepts, develops it as fully as possible (Walker and Avant 2005). This approach aims to select the concept, determine the purposes of analysis, identify all uses of the concept, specify the defining attributes of the concept, construct a model case, create a limit and related case, identify the antecedents and consequences of the concept and define the empirical referents of the concept (Walker and Avant 2005). The details of the literature review will be reported according to the Preferred Reporting Items for Systematic Reviews and Meta‐Analyses (PRISMA) (Page et al. 2021).

### 2.2 Data Collection

Search strategy and information sources: Four electronic databases—Cumulative Index of Nursing and Allied Health Literature (CINAHL; EBSCOhost), MEDLINE (EBSCOhost), Health Management Database (ProQuest), Embase (Elsevier), and Cochrane Library—were consulted in august 2024. A combination of keywords related to the following concepts and associated Medical Subject Heading (MESH) terms was used: ‘nursing care,’ ‘missed nursing care’, ‘emergency department.’ Boolean operators were applied to refine the search and ensure specificity to emergency settings. To ensure relevance, studies published between 2001 and 2024 were included. Studies before 2001 were excluded, as this marked the emergence of ONC as a recognised phenomenon in nursing research. Keywords and Boolean operators are presented in Table 1. Finally, a snowball technique through manual search in the reference lists of eligible studies and in the grey literature was also conducted. In this concept analysis, data extracted from selected studies specifically focused on identifying the conceptual elements of ONC. Unlike traditional literature reviews, here the extracted data explicitly included the attributes (defining characteristics), antecedents (factors contributing to occurrence), consequences (effects on patients and nurses), uses, related concepts, substitute terms, and empirical referents (measures used to identify the concept). Thus, the papers included were reviewed primarily for their contribution to clarifying and delineating these conceptual dimensions, rather than evaluating clinical effectiveness or statistical significance.

**TABLE 1:** Index terms and keywords used in literature research.

Index terms and keywords	
#	Searches
1	‘Emergency service’ or ‘acute care’ or ‘emergency care’ or ‘emergency department*’ or ‘emergency unit*’ or ‘emergency room’ or ‘accident* and emergency department*’ or ‘A&E’
2	Nursing care or emergency nursing or unmet patient need* or missed care or care left undone or rationing of nursing care or ration* care or implicit rationing of nursing care or unfinished nursing care or missed nursing care or error* of omission or delayed care or unmet care need* or omission
3	1 and 2
4	Limit 3 to (yr = “2001–2024”)

Titles and abstracts were screened by a research assistant using the Covidence platform (Systematic Review Software, Veritas Health Innovation, Melbourne, Australia). Titles and abstracts were evaluated to confirm their eligibility for this concept analysis. To be included, studies had to meet the following criteria: (1) To be about ONC or related concepts; (2) by registered nurses (technicians and clinicians); (3) in the context of emergencies (or equivalent environments); (4) based on any qualitative, quantitative, or mixed research design; (5) from 2001 to 2024. Studies involving auxiliary nurses, student nurses and specialised nurse practitioners (NPs) not directly involved in patient care in the emergency setting were excluded as their scope of practice differs from the targeted field.

**FIGURE 1:**
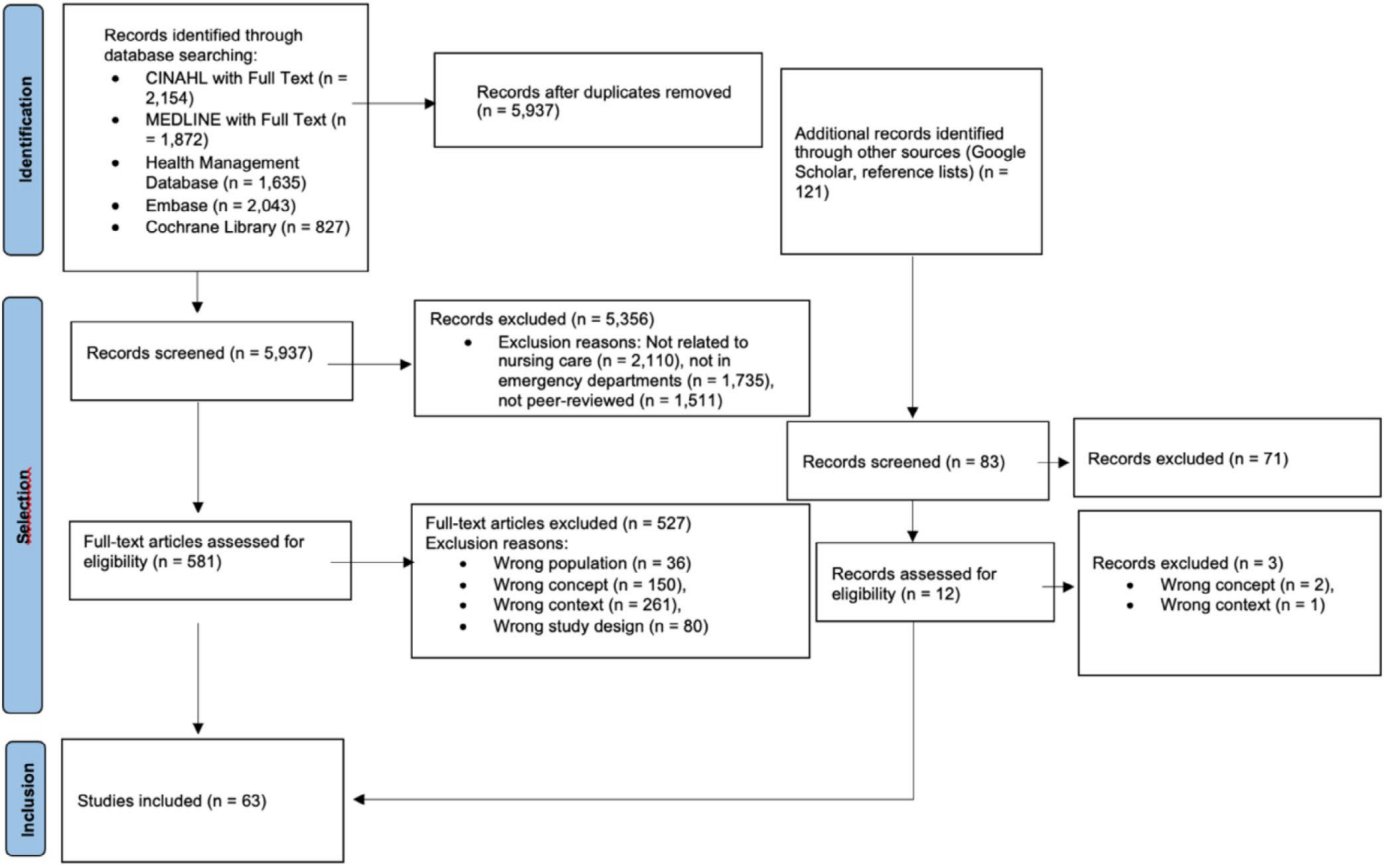
PRISMA diagram.

Research protocols, conference abstracts, knowledge syntheses, and opinion articles were excluded as they are generally not sufficiently detailed and do not present empirical data with enough depth to produce a meaningful synthesis in the context of Walker and Avant's (2005) analysis. The PRISMA diagram detailing the study is shown below (Figure 1).

### 2.3 Data Analysis

Data analysis followed the guidelines of Walker and Avant (2005). Data were synthesised to identify the determining attributes, antecedents, and consequences of ONC in EDs. Special attention was given to qualitative, quantitative, and mixed studies to obtain a comprehensive and nuanced view of the concept. Model cases were developed to concretely illustrate manifestations of ONC in EDs, and empirical referents were identified to measure and evaluate the concept in clinical practice. Details of the studies are presented in Table S1.

## 3 Results

### 3.1 History and Origin of the Concept

The concept of ONC emerged in the scientific literature in the early 2000s, in response to growing concerns about the quality of care in hospital settings. It was particularly highlighted by the works of Kalisch et al. (2009), who defined the concept of “Missed Nursing Care” as the failure to perform necessary nursing tasks due to constraints such as lack of time, staff, or skills. Studies have since shown that ONC is a global problem affecting various care contexts but is particularly pronounced in critical care environments, where time pressure and limited resources are constant challenges (Boltz et al. 2013; Castner et al. 2015).

### 3.2 Uses of the Concept

The uses of the ONC concept include the description and analysis of factors contributing to care omission, evaluation of its impacts on care quality and patient safety, and development of strategies to reduce these omissions. The concept is used to identify gaps in care delivery and guide interventions aimed at improving staffing and organisational practices in emergencies (Boltz et al. 2013; Giles et al. 2019).

To conclude the identification phase of uses, it is essential to determine which use of the ONC concept will be retained for this review. Walker and Avant (2005) emphasise that the researcher must identify whether they wish to consider all uses of the concept or only certain uses. In the context of this work, the use of the concept that will be retained is related to organisational factors and their impact on the omission of care in emergencies. This use is particularly relevant as it helps understand how elements such as the nurses' work environment, staffing and management practices directly influence care quality and patient safety. The studies by Castner et al. (2015) and Giles et al. (2019) clearly demonstrate the importance of these factors in the prevalence of ONC. By focusing on this specific use, it will be possible to build a detailed and targeted portrait of the challenges and potential solutions to reduce ONC in emergency contexts, aligning the objectives of this study with Walker and Avant's (2005) methodological recommendations for a thorough and comprehensive analysis.

### 3.3 Substitute Concepts

The literature review revealed very little information on substitute concepts for ONC. In English, the ONC is referred to as ‘Missed nursing care’ as described by Kalisch et al. (2009). Only two other definitions of the concept were empirically discovered, as depicted in Table 2.

**TABLE 2:** Substitute concepts for the Omission of Nursing Care.

Authors (year)	Terms	Conceptual definitions
Kalisch et al. (2009)	Missed nursing care	Failure to perform or fail to complete necessary nursing tasks due to lack of time, staffing, or skills.
Aiken et al. (2018)	Care left undone	Failing to carry out necessary tasks due to insufficient human resources or inadequate skills.
Schubert et al. (2008)	Implicit rationing of nursing care	Occurs when nurses must make choices about what care to provide due to constraints.

### 3.4 Related Concepts

A preliminary identification of attributes and antecedents to the central concept was initiated through a summary literature review of related concepts, presented in Table 3. Identifying related concepts, as defined, can contribute to identifying and circumscribing the attributes and antecedents associated with the concept of ONC in subsequent steps. Additionally, the related concepts section provides an important foundation for identifying borderline and contrary cases. A borderline case is one where some characteristics of ONC are present, but not to the extent that it fully meets the definition of care omission. For instance, partial or delayed care, rather than complete omission, represents a borderline case. Conversely, a contrary case is one where no defining attributes of ONC exist, such as when all nursing care is provided despite workload constraints.

**TABLE 3:** Related concepts to the Omission of Nursing Care.

Concepts	Links to the omission of nursing care
Nursing workload	Excessive workload can contribute to ONC due to lack of available time or resources (Tubbs‐Cooley et al. 2019).
Patient security	ONC can compromise patient safety by exposing patients to increased risks of sentinel events (Rochefort et al. 2016).
Quality of care	ONC has a direct impact on the quality of care provided (Chaboyer et al. 2021).
Management of health human resources	Staffing policies, nurse–patient ratios, and management practices can influence the likelihood of omission (Rochefort and Clarke 2010; Rochefort et al. 2016).
Organisational culture	Organisational values, norms, and practices can influence nurses' behaviour and their propensity to perform or omit care (Jones et al. 2015).
Nursing education and skills	Role in preventing neglect of care by strengthening the capacities of nursing staff (Burnes Bolton et al. 2007).
Technology	Facilitates the coordination of care and reduces the risks of care omission by allowing effective monitoring of tasks carried out (Stemmer et al. 2022).
Nursing monitoring	Mechanisms and processes that help prevent omission by identifying priority tasks and ensuring they are completed on time (Jones et al. 2015).

#### 3.4.1 Identification of Determining Attributes of ONC

The determining attributes of ONC in emergency departments can be characterised by several key elements. These attributes include the incompletion of essential care, the interruption or delay of priority nursing interventions, and the failure to provide necessary care within the required timeframe. These elements are intrinsic to the nature of ONC and define the phenomenon itself, rather than simply contributing to its occurrence.

First, incompletion of essential care is a fundamental attribute. This occurs when nurses are unable to deliver key aspects of care that are crucial for patient safety and recovery, such as the administration of prescribed medications, continuous monitoring of vital signs, or necessary patient assessments (Boltz et al. 2013). This incompletion represents a direct omission of care that compromises patient outcomes.

Another defining attribute is the delay or interruption of priority interventions. In emergencies, timely interventions are crucial for the stabilisation of patients. Any delay or interruption in delivering these interventions, such as the rapid assessment of critically ill patients or the administration of life‐saving treatments, constitutes an omission of care (Mitchell Scott et al. 2014).

Finally, failure to provide necessary care within the required timeframe is an essential attribute of ONC. Emergency care demands swift, decisive action, and the inability to deliver care within an appropriate timeframe can be fatal. This includes situations where patients do not receive timely interventions due to competing priorities, or where nurses cannot prioritise effectively due to high patient volumes (Giles et al. 2019).

Classically, the ONC can be defined as the failure to deliver essential care within the required timeframe, under the high‐pressure and unpredictable nature of ED settings, resulting in the incompletion or interruption of priority interventions that are critical to patient safety and care quality.

### 3.5 Identification of Model Cases

The identification of model cases is crucial in Walker and Avant's method as it concretely illustrates the concept of ONC in the emergency department, making its attributes and manifestations more tangible and understandable. Moreover, these model cases serve as references to distinguish ONC from similar phenomena, facilitating the precise clarification and delimitation of this concept in the specific context of emergencies. The two cases below are derived from real‐life experiences during my first year as a nurse.

Case 1 Emergency department during peak hours.

In an urban hospital emergency department, a nurse must simultaneously manage five patients with critical conditions. Due to a lack of staff, she omits to check a patient's vital signs, leading to the patient's condition deteriorating. This situation illustrates how excessive workload and lack of human resources lead to care omissions (Boltz et al. 2013).

Case 2 Rural setting with limited resources.

In a small rural emergency department, the nurse is responsible for several administrative tasks in addition to her clinical responsibilities. Due to inadequate medical equipment (equipment malfunction), she cannot perform an intravenous infusion for a dehydrated patient, delaying the patient's treatment. This case shows how lack of resources and extensive responsibilities can lead to care omissions (Castner et al. 2015).

Case 3 Night shift with high patient turnover.

During a night shift in a busy emergency department, a nurse is responsible for newly admitted patients and those awaiting transfer to inpatient units. Due to a high turnover and administrative workload, she is unable to reassess a patient's pain level after administering an analgesic. The patient continues to experience unrelieved pain for several hours. This case highlights how administrative burden and continuous patient flow contribute to ONC, particularly in pain management and patient comfort.

### 3.6 Identification of Additional Cases

#### 3.6.1 Limit Case

In the emergency department, the nurse performs all necessary interventions except administering a non‐urgent medication (cholesterol management) due to a moment of high influx in the department. Although this care was omitted, it did not result in severe consequences for the patient. This limit case illustrates an omission without immediate severe impact on the patient's health (Mitchell Scott et al. 2014).

#### 3.6.2 Related Case

In a paediatric emergency unit, the nurse observes partial care practices, where some interventions are deliberately postponed, such as oral care for a patient. Although this does not constitute a strict omission, it can be related to the issue of partial or fragmented care (Giles et al. 2019).

### 3.7 Identification of Antecedents and Consequences

#### 3.7.1 Antecedents

Understaffing: A key antecedent of ONC in EDs, where insufficient nurse‐to‐patient ratios increase the likelihood of missed care (Castner et al. 2015).

Inadequate training and competency gaps: Nurses lacking specific skills or training for critical interventions are more likely to omit necessary care (Boltz et al. 2013).

High patient volume and overcrowding: The unpredictable influx of critically ill patients in EDs leads to task overload, forcing nurses to prioritise acute cases at the expense of others (Fleet et al. 2019).

Time pressure and workload intensity: Emergency nurses frequently experience time constraints that impede the completion of all necessary care tasks, increasing the risk of ONC (Recio‐Saucedo et al. 2018).

Lack of standardised protocols and clinical guidelines: Inconsistent or unclear policies for triage, pain management, and documentation increase the likelihood of ONC (Aiken et al. 2018).

Limited access to essential medical resources: The absence of vital equipment, medications, or supplies can hinder the provision of timely and complete nursing care (Burnes Bolton et al. 2007).

Poor interprofessional collaboration: Ineffective communication and teamwork between nurses, physicians, and other healthcare professionals contribute to care fragmentation and omissions (Duhalde et al. 2023).

Frequent interruptions and multitasking demands: Nurses working in EDs must often manage multiple patients simultaneously, leading to task‐switching and potential care omissions (Chaboyer et al. 2021).

Moral distress and ethical dilemmas: Nurses experiencing moral distress due to conflicting professional responsibilities may choose to omit non‐urgent care tasks to focus on immediate life‐threatening cases (Stemmer et al. 2022).

Fatigue and shift work‐related stress: Long shifts, night work, and irregular schedules reduce cognitive function and decision‐making abilities, increasing the likelihood of ONC (Ball et al. 2014).

Limited nurse autonomy and decision‐making power: Institutional constraints, including hierarchical decision‐making processes, may prevent nurses from taking timely action to address patient needs (Kalisch et al. 2009).

Lack of leadership and managerial support: Inadequate supervision, mentorship, and lack of support from nurse managers contribute to a culture where ONC becomes normalised (Aiken et al. 2018).

Psychological and emotional burden: Repeated exposure to trauma, patient deaths, and high‐acuity cases can lead to emotional exhaustion and compassion fatigue, reducing nurses' ability to provide comprehensive care (Papathanasiou et al. 2024).

#### 3.7.2 Consequences

Patient complications: ONC in EDs leads to increased sentinel events, morbidity, and mortality (Mitchell Scott et al. 2014).

Nurse burnout: Repeated ONC contributes to nurse dissatisfaction and burnout, exacerbating staff shortages (Giles et al. 2019).

Increased patient morbidity and mortality: ONC in EDs leads to higher rates of complications, including preventable infections, sepsis, delayed diagnosis, and deterioration of patient conditions (Mitchell Scott et al. 2014).

Higher incidence of adverse events: Missed nursing care is strongly associated with medication errors, pressure ulcers, falls, delayed wound healing, and pain mismanagement (Recio‐Saucedo et al. 2018).

Prolonged hospital stays and increased readmission rates: Patients experiencing ONC in EDs may require longer hospitalisations or return to the ED due to unresolved or worsening conditions (Rochefort et al. 2016; Sasso et al. 2017).

Compromised patient satisfaction and trust in healthcare: Delays, incomplete care, and insufficient communication contribute to patient dissatisfaction and reduced confidence in emergency services (Jones et al. 2015).

Increased emergency department congestion: ONC can lead to care delays that result in extended ED wait times, exacerbating overcrowding and reducing system efficiency (Fleet et al. 2019).

Nurse burnout and job dissatisfaction: Repeated ONC leads to emotional exhaustion, decreased professional fulfilment, and higher attrition rates among emergency nurses (Giles et al. 2019).

Moral distress and ethical conflicts among nurses: Witnessing or participating in ONC can create internal conflicts for nurses who feel unable to meet professional standards, increasing emotional distress and intent to leave the profession (Stemmer et al. 2022).

Higher turnover rates and staffing shortages: Chronic ONC contributes to increased resignation rates among emergency nurses, exacerbating the staffing crisis in healthcare systems (Aiken et al. 2018).

Financial burden on healthcare systems: ONC leads to higher healthcare costs due to preventable complications, increased interventions, and resource utilisation (Jones et al. 2015; Sasso et al. 2017).

**FIGURE 2:**
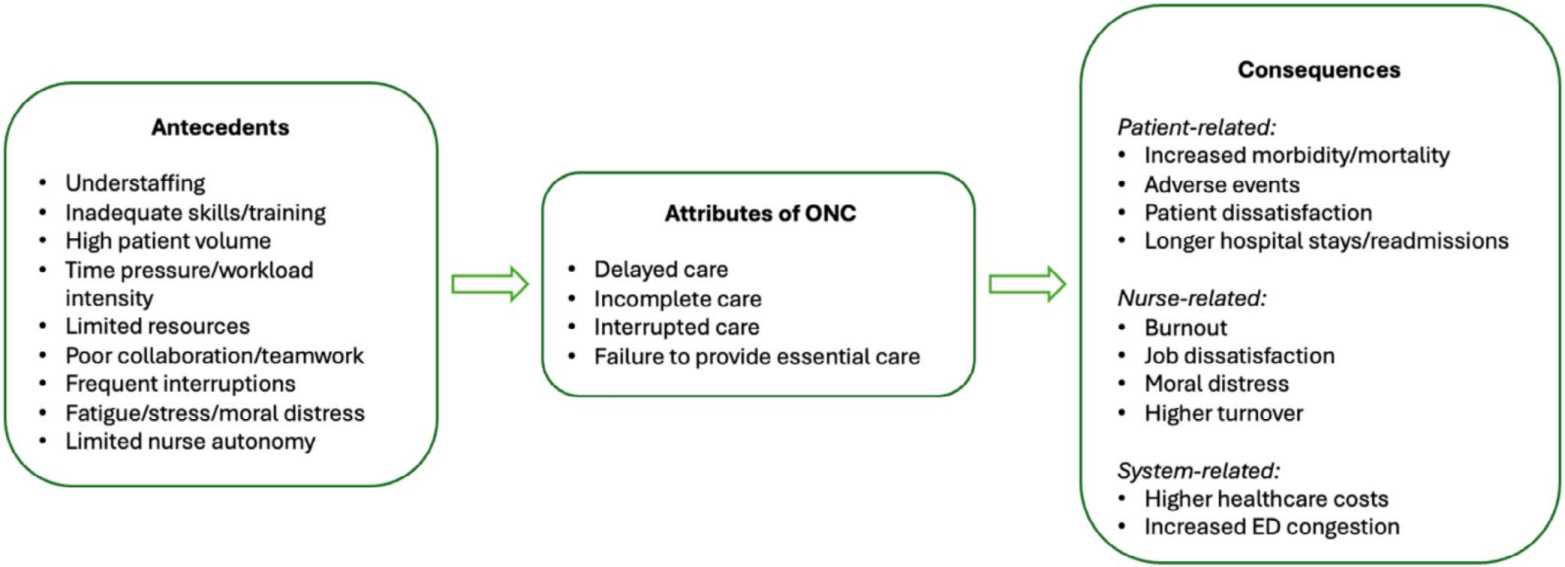
Conceptual framework of ONC in EDs.

Figure 2 visually summarises the conceptual framework developed through this analysis, illustrating clearly the relationships between antecedents, attributes, and consequences of the omission of nursing care within emergency department contexts.

#### 3.7.3 Identification of Empirical Referents and Existing Measurement Instruments

To measure ONC in EDs, it is crucial to identify empirical referents that allow for precise and practical evaluation of the concept. These empirical referents must be based on validated measurement tools and robust methods to ensure the reliability and validity of collected data. Among the available instruments, the Missed Nursing Care Survey (MISSCARE) is the most widely used and specifically designed tool to assess ONC. Other measures, such as the Basel Extent of Rationing of Nursing Care (BERNCA) and the Task Left Undone (TLU) scale, provide insights into rationed care or incomplete tasks but do not directly measure ONC in the same way. Most other measures used qualitative methods or focused on broader aspects of care quality and structural variables rather than explicitly identifying omitted nursing care.

The MISSCARE is the most validated and frequently utilised tool to measure the omission of nursing care. Developed by Kalisch et al. (2009), this questionnaire assesses the frequency and reasons for missed nursing care. It includes questions on various nursing tasks and identifies areas where care is often omitted. This tool has been widely validated and used in various international studies, including those focusing on emergency services (Castner et al. 2015; Palese et al. 2016). However, no existing instrument, including the MISSCARE, has been specifically contextualised to the emergency department setting. The fast‐paced, unpredictable nature of EDs requires an adapted measurement tool that accounts for rapid patient turnover, triage‐based decision‐making, and the unique challenges of emergency nursing care. While the MISSCARE remains the most comprehensive tool available, it does not fully capture the complexity of ONC in emergency contexts. Developing an ED‐specific measurement instrument remains a critical gap in ONC research.

## 4 Discussion

In this study, Walker and Avant's method was applied to analyse the concept of ONC in EDs. The analysis revealed that ONC in EDs, while sharing similarities with general concepts of care omission, is distinctly shaped by the specific constraints of emergency care. These constraints include the unpredictability of patient flow, the critical and often unstable conditions of patients, and the requirement for rapid decision‐making and interventions. Unlike general healthcare rationing, which focuses on the distribution of scarce resources, ONC in EDs is characterised by the unintended failure to provide essential care due to the combined effect of systemic and organisational limitations (Kalisch et al. 2009; Schubert et al. 2008).

Several key antecedents contribute to ONC in EDs. A primary antecedent is the high‐pressure environment typical of emergency departments, where the unpredictable nature of emergencies and the constant influx of patients create excessive workloads (Fleet et al. 2019). This unique environment can result in the omission of critical nursing tasks, such as patient monitoring and timely medication administration, which are vital for ensuring patient safety (Recio‐Saucedo et al. 2018). Additionally, staffing shortages and inadequate skill mixes exacerbate these pressures, forcing nurses to prioritise certain tasks over others, often leading to care omissions (Jones et al. 2015).

The varying complexity and urgency of patient cases in EDs also distinguish the context of ONC from other settings. In EDs, nurses must frequently switch between stabilising patients in critical condition and managing less urgent cases. This constant shift in priorities increases the likelihood of care omissions, particularly when resources are strained. Populations such as paediatric patients or older adults with multiple comorbidities present additional challenges, as they often require more comprehensive and individualised care, which can be overlooked during busy periods. Notably, paediatric patients—a significant population in many EDs—are particularly vulnerable to care omissions due to their unique care needs and developmental considerations. Despite this, there is a notable absence of measurement tools specifically adapted to capture ONC in paediatric populations. Current instruments, such as the MISSCARE, are designed for general adult populations and do not fully address the distinct developmental, emotional, and physiological needs of children in emergency settings. This gap in measurement poses a significant challenge in assessing the true scope of ONC among paediatric patients, an issue that is increasingly relevant given the diverse age groups and conditions treated in EDs.

ONC in EDs has significant consequences for patient safety and care quality. Higher rates of ONC are associated with increased morbidity and mortality, as well as a rise in adverse events such as medication errors, infections, and falls (Aiken et al. 2018; Recio‐Saucedo et al. 2018). The financial implications are also substantial, as ONC leads to longer hospital stays and higher readmission rates, which in turn increase overall healthcare costs (Jones et al. 2015; Sasso et al. 2017). For nurses, the persistent occurrence of ONC contributes to job dissatisfaction, burnout, and moral distress, resulting in higher turnover rates (Ball et al. 2014; Stemmer et al. 2022).

To measure ONC, the MISSCARE is the most widely validated tool. However, while it provides valuable insights into the frequency and reasons for care omissions, this tool is not fully adapted to the unique urgency and specificity of the ED context. The fast‐paced and unpredictable environment of EDs requires more targeted measurement tools that can capture the nuances of care omissions in this setting (Ausserhofer et al. 2014; Kalisch et al. 2009).

Understanding the attributes, antecedents, and consequences of ONC in EDs is essential for developing interventions to mitigate its impact. To reduce ONC, healthcare institutions should prioritise evidence‐based staffing models, such as dynamic nurse–patient ratios that adapt to patient acuity and ED occupancy (Rochefort et al. 2016). This could involve real‐time patient flow monitoring systems that trigger automatic staffing adjustments during peak admission hours. In addition to staffing improvements, the integration of emergency nurse training programmes focused on triage efficiency, rapid decision‐making, and crisis management could better prepare nurses to manage workload surges without compromising essential care tasks (Charette et al. 2023; Tessier et al. 2023). Additionally, AI‐driven decision support systems could be implemented to assist nurses in task prioritisation and reduce cognitive overload in fast‐paced ED environments. From a policy standpoint, hospitals should establish mandatory ONC reporting frameworks, where instances of missed care are systematically documented and analysed to develop targeted quality improvement initiatives. Furthermore, creating a supportive work culture that encourages nurses to report ONC without fear of reprisal is critical for identifying systemic issues and implementing corrective measures.

Moreover, empirical research that examines the effectiveness of these interventions in EDs is necessary. Future studies should also consider diverse healthcare settings and cultural contexts to provide a more comprehensive understanding of ONC. A comparative analysis of ONC rates in different ED models, such as standalone emergency centres versus large urban trauma centres, could further identify structural factors influencing ONC. By enhancing our understanding of ONC through this conceptual analysis, healthcare professionals can improve patient safety, optimise care quality, and better support nursing staff in high‐stakes environments.

## 5 Study Limitation

This concept analysis has several limitations that need to be acknowledged. Firstly, the scope of the literature review was constrained by the availability of databases accessible to the researchers. This limitation may have resulted in the exclusion of relevant studies published in non‐accessible databases, potentially introducing a selection bias. Additionally, the review focused solely on English and French articles, which may have excluded significant contributions from studies published in other languages.

Another limitation is the potential variability in the definitions and measurements of ONC across different studies. Although efforts were made to standardise the conceptual framework, variations in operational definitions and measurement tools may affect the comparability of findings. The reliance on self‐reported data in many of the included studies also introduces the risk of response bias, as nurses may underreport or overreport instances of ONC.

Furthermore, the study's focus on EDs may limit the generalisability of the findings to other healthcare settings. While the high‐pressure environment of EDs presents unique challenges, the factors contributing to ONC in other contexts, such as medical‐surgical units or long‐term care facilities, may differ. Future research should explore ONC across diverse healthcare settings to provide a more comprehensive understanding of the phenomenon.

Lastly, the rapidly evolving healthcare landscape, influenced by changes in policies, technologies, and workforce dynamics, means that the findings of this analysis may not fully capture the current state of ONC. Continuous updates to the conceptual frameworks are necessary to keep pace with these changes and to ensure the relevance of interventions aimed at reducing ONC.

## 6 Conclusion

This concept analysis provides a foundation for targeted practice, policy, and research interventions aimed at reducing ONC in EDs. To improve clinical practice, healthcare institutions must adopt evidence‐based staffing strategies that dynamically adjust nurse‐to‐patient ratios based on real‐time patient acuity and departmental workload. Introducing advanced training programmes focused on prioritisation and rapid decision‐making skills can also equip nurses to manage workloads more effectively, mitigating risks of care omissions. From a policy perspective, healthcare leaders should prioritise creating standardised reporting mechanisms for ONC incidents. Establishing mandatory, non‐punitive reporting frameworks will facilitate the collection of reliable data, enabling health systems to develop targeted quality improvement initiatives. Policies should also foster supportive workplace cultures, empowering nurses to openly report care omissions and actively participate in solutions, thereby enhancing patient safety and nurse retention.

Further research should concentrate on developing specialised tools tailored explicitly for measuring ONC in paediatric emergency settings, addressing a critical gap in the current literature. Additionally, comparative studies evaluating ONC interventions across diverse health systems and cultural contexts will enhance understanding and inform international policy and practice standards. By addressing these implications, healthcare organisations globally can significantly improve patient outcomes, strengthen nursing workforce stability, and enhance the overall quality of emergency care.

## Conflicts of Interest

The authors declare no conflicts of interest.

## Supporting information


Table S1.

## Data Availability

The authors have nothing to report.
