# Supplementary material for: The Omission of Nursing Care in Emergency Departments: A Conceptual Analysis Using Walker & Avant's Methodology
Source: J Adv Nurs. 2025 May 1;82(2):1750–9. doi: 10.1111/jan.17017 (PMC12810661; doi:10.1111/jan.17017)
Supplement: Supplementary file 1 — Table S1. [file JAN-82-1750-s001.docx]

Supplementary table 1

**Table 1.**

Included studies (n = 63).

| **Authors/Year/Country** | **Study purpose** | **Design** | **Data collection instruments and procedures** | **Sample & setting** | **Key findings** |
| --- | --- | --- | --- | --- | --- |
| Boltz, M., Parker, B., Shuluk, J., Capezuti, E., & Galvin, E, J. (2013).  USA. | To describe nurses’ views of the issues to be addressed to improve care of the older adult in the emergency department (ED). | National survey. | A secondary analysis of the GIAP survey/instrument 157- item, anonymous, self-report tool supplemented with open-ended question. | 527 responses. The nurses reported 15.8 (±9.4) mean years of experience as a nurse. The proportion of basic education was as follows: associate degree (n = 187; 35.5%), bachelor’s degree (n = 183; 34.7%), and diploma preparation (n = 105; 19.9%) and masters’ degree (n = 52; 9.9%).  49 acute care hospitals in the United States. Academic medical center, Teaching hospital, non-teaching hospital. Hospital size: Small, medium, and large. | The nurses’ descriptions of the pressing issues surrounding care of older adults in the ED were lack of time to care for older people with complex multisystem issues and competing demands leading to shortcomings in preventive care, teaching, poor communication and psychosocial support. This could in turn lead to missed adverse reactions, pressure ulcers, falls and urinary tract infections. |
| Giles, T., Hammad, K., Breaden, K., Drummond, C., Bradley, S., Gerace, A., & Muir-Cochrane, E. (2019). Australia. | To explore nurses’ perceptions and experiences of caring for patients who die suddenly and unexpectedly in an ED setting. | A qualitative descriptive approach. | Open-ended questions from an online survey. | 211 ED nurses.  Australian EDs located at major regional, metropolitan, or tertiary referral hospitals. | ED nurses want to provide high quality care to dying patients and their families and recognize the harmful effects on everyone involved when this does not occur. However, multiple barriers exist which jeopardies this care. These were: Overcrowding, time pressure, competing priorities, staffing constraints, national emergency access targets, bed block, and inappropriate admissions from aged-care facilities. Critically ill patients were often prioritized over dying patients due to inadequate staffing ratios. Nurses expressed that this affected their abilities of providing effective EOL care, dignified end of life strategy in the ED. |
| Mitchell-Scott, B., Considine, J., & Botti, M. (2014). Australia. | To determine the frequency and nature of medication errors in emergency care. | Prospective, exploratory descriptive design. | Study specific point prevalence survey (Patient demographics and ED characteristics, Medication administration variables).  Nine-point prevalence surveys were conducted during 2009. | 172 patients treated in the ED cubicles during the point prevalence study were included.  One Setting: ED, 61700 attendances per year. | Medication errors related to patient identification, allergy status and medication omissions occur more frequently when the ED is busy, has sicker patients and when the staffing is not at the minimum required staffing levels. |
| Castner, J., & Dean-Baar, S. (2015). USA. | To build and test a multi-level model on the contextual relationships and interactions of individual RN and nursing unit factors on missed nursing care. | Exploratory descriptive cross-sectional design. | MISSCARE Survey.  Survey packets were distributed in December 2011 and January 2012. | A convenience sample of all RNs in direct patient care or unit-level management. 5.1% ED nurses (n = 28) of 2509 RNs from five hospitals working in 35 different units. 553 surveys retained.  Northeastern United States, five-hospital system. | The amount of missed nursing care in this study demonstrates a significant risk to the quality and safety of hospital patient care. Nurses are routinely unable to complete care to basic standards, leaving patients vulnerable. Aggregated by nursing unit (Medical-surgical, critical care, peri-operative/interventional, obstetric, emergency, rehabilitation) the highest single unit missed nursing care score was 3.20 (SD = 0.47) in an emergency department. Emergency (M = 2.74, SD = 0.60) units demonstrated the highest amount of missed nursing care. |
| Honan, B., Davoren, M., Preddy., & Danieletto, S. (2020). Australia. | To describe pain assessment and management for patients in a regional Australian ED with a hip fracture, compared to national guidelines and recommendations as well as the performance of other Australian EDs as reported by the ANZHFR. | Retrospective single-site observational study. | For all participants, data was electronically extracted from the data warehouse on diagnosis, age, residential postcode, arrival and departure time and date, triage category and length of stay in ED.  Data was manually extracted from paper-based medication charts and the electronic medical record on initial and final pain scores, time of documentation of pain score, analgesia and/or nerve block administered prior to arrival in the base hospital and within the ED of the base hospital. | The participant population was all patients attending the ED from 1 July 2017 to 31 December 2017. A patient was eligible for inclusion if they were aged 18 years or over and had the following type of fracture: intracapsular, extracapsular, trochanteric or subtrochanteric. Patients were excluded from analysis where there was no electronic or paper-based record available. 93 records were analyzed.  A regional referral center that sees 42000 presentations to the ED annually. | Potential targets for quality improvement in the management of pain for hip fracture patients in the ED included improving the documentation of pain scores within 30 min of arrival and administering a nerve block as a key component of analgesia. Pain assessments was not documented or done in 24%. |
| Lee, S., Hong, H., Choi, M., & Yoon, J. (2021). South Korea. | To examine whether ED nurses’ workload led to negative consequences (nurses’ insufficient compliance to pain management policies) and outcomes (inadequate analgesia) in the ED. | Retrospective correlational study. | Gender, age, vital signs, and acuity were collected through the clinical data warehouse. Pain scores were confirmed through nursing records. Nurses’ schedules and personnel data were used to examine nursing staff factors, and the name of the analgesics and the time taken to administer them were verified through the doctors’ prescription records. According to the study institution’s guidelines, pain should be assessed initially at triage and reassessed within 1 h of administering analgesics. | Inclusion criteria were patients who 1) visited the ED from Jan. 1 to Dec. 31, 2019 for abdominal pain, 2) aged 18 or older, 3) had an NRS 4 or above on arrival, and 4) were administered analgesics. A total of 1428 cases were included in the final analysis.  Tertiary hospital with an average annual number of ED visits of about 70000 patients. | We identified a significant association between nursing staff factors and the time to administration of analgesics and pain reassessment. The time from prescription to administration increased as the nurse-to- patient ratio increased, but the time was not affected by the ED experience of nurses. Conversely, the nurse-to-patient ratio at the time of prescription was not associated with pain reassessment, but nurses with more ED experience were more likely to reassess the patients’ pain. |
| Wagner, J., MacPhee, M., Udod, S., Berry, L., Perchie., & Conway, A. (2021). Canada. | To investigate the impact of a patients’ needs assessment (synergy tool) on emergency department nurses’ perceptions of quality, safe care delivery and morale. | Quantitative descriptive. | The survey included eight demographic questions, one intent- to-leave question (Kalisch et al., 2010), six workload/staffing questions (Hospital Survey on Patient Safety Culture, 2021; Lake, 2002), four spirit-at-work questions (Kinjerski, 2013; Wagner et al., 2013), eight quality/safety questions (Sermeus et al., 2011).  Surveys were administered seven months before introducing the synergy tool to emergency department nurses and 15 months after implementation. The survey was administered electronically | The sample population consisted of the total population of emergency department nurses, registered nurses licensed practical nurses. A total of 158 respondents. Eighty-seven per cent of the sample were registered nurses. Data from 8841 shifts were included.  Two emergency departments. | Increases in patient volume and patient acuity may lead to significant changes in ED nurse workloads, creating concerns regarding the individual nurse's ability to provide care. Critical patient care may be missed during periods of ED overload, placing patient and staff at risk, ultimately leading to reduced nurse morale, described as spirit at work in this study and an increase in ED nurses’ intent to leave. |
| Senek, M., Robertson, S., Ryan, T., King, R., Wood, E., & Tod, A. (2020). United Kingdome. | To consider various acute care settings in order to explore the interaction effect between permanent, agency staffing ratios and ‘care left undone’. | Cross-sectional study. | Online survey of registered nurses (RNs) developed and administered by The Royal College of Nursing (RCN).  Data was collected between the 14th May and 30th May 2017. The survey was administered via email and social media. | The focus of this research was on the adult acute setting and the final findings comprised responses relating to 13218 (ED = 7124) staff who worked in this sector. The respondents identified pre- dominantly as ‘staff nurse’ (71.8%, N = 9490), 22.4% (N = 2960) identified as sister/charge nurse, 3% (N = 397) as clinical nurse specialist and 2.9% (N = 396) as senior nurse. We included RNs working in Emergency Department (ED), Adult Acute, Critical Care, Older People’s ward and Theatre and was open to both members and non- members of the RCN. | These findings suggest a worrying prevalence of under-staffing and care left undone in UK acute care sector nursing. The highest proportion of “care left undone” was within the ED setting (48.4%) (Adult Acute Ward (45,3%) Critical Care/ high dependency (27,7%) Older people’s ward (46%) and Theatre (21%). |
| Alsharawneh, A., Maddigan, J., Gaudine, J., Etchegary, H., & Gao, Z. (2020). Canada. | To evaluate the quality of ED care of patients with FN in terms of 3 quality dimensions: safety, effectiveness, and timeliness of care. | Retrospective observational design. | Data from the cancer registry were transferred to the province’s health information agency. Registry data were linked with the identified hospital’s ED patient information. Data were collected by conducting chart reviews using a standardized chart review form (CRF). ED quality of care was evaluated in terms of three quality dimensions: safety, effectiveness, and timeliness of care (part of the Canadian ED national benchmarks). | Targeted population included all cancer patients in the provincial cancer registry with a first presentation of fever to 1 emergency department that occurred on a date after the time of their cancer diagnosis. Patients (n = 431) were included in the study if they were (1) aged at least 18 years, (2) undergoing active cancer treatment (were on chemotherapy treatment within a month of ED presentation), and (3) came to the emergency department with a presentation of fever between April 1, 2011, and March 31, 2016.  One urban emergency department. | Most of the patients who waited at triage were not reassessed by the triage nurse at the intervals recommended in the CTAS guidelines. On average, patients had to wait for 228 minutes before the administration of antibiotics, which was significantly longer than the benchmark of 60 minutes. Only 4.0% of the patients in our sample received antibiotics within the time frame of 1 hour as recommended in the FN guidelines. |
| White-Trevino, K., & Dearmon, V. (2018). USA. | To describes the QI process undertaken to implement a structured, patient- centered report process in the ED. | Quality improvement project. | Data was collected via observation of handoff using an SBAR-T competency checklist, a two-question study specific survey and the Press Ganey Emergency department survey. Observations proceeded for 12 weeks.  The survey was conducted one month following implementation of the new report system. Patient satisfaction with nurse communication was assessed five nurse indicators pre and post innervation. All handoffs were observed at the 7:00 PM shift change on five different evenings during the three-month evaluation period. | Participants consisted of 46 emergency registered nurses. Thirteen handoffs were observed, with 12 (92%) of these occurring at the bedside.  One hospital based ED. | Patients report satisfaction with the bedside report and with nurse communication when the structured report process is moved to the bedside, and the patient is engaged in the process. Nurses perceive that the bedside report process heightens their ability to attend to patient needs and to listen to patients. However, nurses failed to consistently validate the patient armband, assuming the patient identity as it was reported by off going nurses. |
| Scott, B., Constine, J., & Botti. (2015). Australia. | To determine the frequency and nature of unreported clinical deterioration in emergency care. | Prospective, exploratory descriptive design. | Study specific point prevalence surveys (PPS). PPS were used to collect data related to unreported clinical deterioration. In addition, data related to contextual factors such as ED patient characteristics, staffing and workload were also collected during the PPS.  A total of nine PPS was conducted between 1 May and 30 June 2009 and occurred over various days of the week and times of the day and night. All ED cubicles were included in each PPS. | All ED patients (n = 186) receiving care in an ED cubicle during the times of the PPS were included in the study: patients in the waiting room were excluded from the study.  The study was conducted in the northern suburbs of Melbourne, Australia. When the study was conducted, the ED was managing approximately 61700 attendances per year. | The results of this study show that the age and clinical urgency of the whole ED patient cohort influenced the prevalence, 12.9%, of unreported clinical deterioration. ED occupancy also influenced the prevalence of unreported clinical deterioration. While there is evidence regarding the adverse effects of ED overcrowding, the effects of different levels of ED occupancy in specific patient care areas of the ED warrant further investigation. |
| Kerr, D., Klim, S., Kelly, A-M., & McCann, T. (2016). Australia. | To evaluate whether implementation of a new nursing handover model resulted in improved completion of nursing care activities and documentation. | A pre- and post-implementation study. | Clinical Handover Staff Survey. Direct observation Audit data were collected, on an explicit data form. Clinical Handover Staff Survey’ was adapted for the study to better fit the ED setting.  Survey data were collected across two distinct five day phases: pre-implementation (pre) February 2011, post- implementation (post) December 2011. Audit data were collected across two distinct five day phases: pre-implementation (pre) June 2011, post- implementation (post) December 2011. | A convenience sample of nurses working in the department completed the survey. Eligible participants included all permanent and casual nursing staff employed on any shift during the designated 5-day data collection periods. A total of 126 surveys were completed in the two study periods: pre (n = 67) and post (n = 59). A total of 368 medical records and patient observations were audited in the two study periods in the ED: 173 in the pre-intervention phase and 195 in the post- intervention phase.  The study was conducted in a mixed adult and pediatric ED of a teaching hospital in Melbourne, Victoria, Australia. | The ED structured nursing handover framework focused on a standardized approach, including checklists, with emphasis on nursing care and patient involvement. Patients with allergies to medication were more likely to be wearing an allergy alert band and patients were more likely to be wearing an identification bracelet. Finally, increased rates of documentation were observed for intravenous cannula insertion, and intravenous therapy recorded on the fluid balance chart. Respondents were less likely to report that ‘important vital sign observations are often omitted from nursing handover. |

| **Authors/Year/Country** | **Study purpose** | **Design** | **Data collection and instruments** | **Sample & setting** | **Key findings** |
| --- | --- | --- | --- | --- | --- |
| Wolf, L., Delao, A., Malsch, A., Moon, M., Perry, A., & Zavotsky, E. (2019).  USA. | To explore emergency nurses’ perceptions of their ability to care for geriatric patients in the emergency department, including identification of facilitators and barriers to safe care. | Mixed-methods sequential design. | Modified Pennsylvania Emergency Department Geriatric Readiness Survey and semi structured interview.  Survey data distributed via email from the membership of a large nonprofit organization and collected before the focus groups, allowing further exploration and clarification through the focus-group discussion. Focus-group participants were recruited by e-mail from a list of emergency nurses registered to attend a conference in September 2018. Focus-group sessions lasted approximately 1 hour each, were audio recorded. | A nationally representative sample of English-speaking emergency nurses above the age of 18 years was recruited for the survey (N 1⁄4 1610). The two focus group samples comprised 23 emergency nurses who worked in the United States (86%), Canada (4.7%), and Hong Kong (9.5%).  Emergency setting. | Identified barriers to improved care include a lack of integration between emergency care and community care, deficits in geriatric-specific education, inconsistent use of early screening for frailty, and lack of resources in the emergency care environment to intervene appropriately. |
| Cetin-Sahin, D., Ducharme, F., McCusker, J., Veillette, N., Cossette, S., Minh Vu, T., Vadeboncoeur, A., Lachance, P-A., Mah, R., & Berthelot, S. (2021).  Canada. | To explore experiences of an ED visit among patients aged 75 and older. | Mixed method. Qualitative descriptive design. | Data retrieved from ED registries: Patient sex, age, autonomy code (ambulatory vs bed), the Canadian Triage and Acuity Scale. Telephone survey included: open-ended structured questionnaire comprising 26 items representing potential problematic experiences.  During weekday work hours, 3 trained research assistants (RAs) identified patients aged 75 years and older in ED clinical registries. Potentially eligible patients or proxies signed informed consent forms and completed a face-to-face baseline questionnaire in the ED. RAs conducted telephone interviews with participants 1 week after discharge. | A cohort of patients aged 75 and older discharged to their original residence (own home, residence, nursing home) was recruited from July 2014 to February 2016. We targeted patients aged 75. Of 843 eligible patients contacted in the ED by an RA, 481 patients or their proxies provided written consent. Of the 481 who consented, 412 completed the 1-week interview. Among 108 selected participants, 32 family members (26 children, 5 spouses, and 1 friend) completed the interviews on behalf of patients.  Four university affiliated EDs in two cities Total number of annual visits ED 1. 38124. ED 2: 78814, ED 3: 74305. ED 4: 30310. | Despite the existence of evidence-based geriatric ED guidelines, in our sample of 4 hospitals, patient and family experiences were often inconsistent with these recommendations. Our findings reinforced the fact that, locally, each ED could improve their care of older adults, particularly those who are unable to communicate and/or are not mobile. More emphasis is needed on preparation for the transition back home; this could be accomplished through appropriate patient education and collaboration with community-based care providers—the family doctor, com- munity pharmacist, and home care services. |

| **Authors/Year/Country** | **Study purpose** | **Design** | **Data collection and instruments** | **Sample & setting** | **Key findings** |
| --- | --- | --- | --- | --- | --- |
| Olofsson, P., Carlström, E., & Bäck-Pettersson, S. (2012). Sweden. | To describe and understand chronically ill elderly patients’ experiences during their ED stay. | Inductive qualitative approach. | Descriptive phenomenological method Giorgi’s (2009).  Individual interview, open ended questions. | 14 patients (> 70 years old), > 3 clinical diagnoses, > 3 ED visits during the last 12 months, yellow or green priority.    One Swedish ED. | The visit to the ED was experienced as contradictory. The triage encounter fostered confidence and set promising expectations, but during the rest of the visit, the patient felt abandoned and considered the staff to be uncommitted and reluctant. Basic needs were not cared for, e.g., lack of food, analgesia, and information. This could lead to persistent negative associations to the ED. |
| Gallagher, R., Fry, M., Chenoweth, L., Gallagher, P., & Stein-Parby, J. (2014). Australia. | To investigate ED nurses’ perceptions and experiences of providing care for older people. | Qualitative design. | Thematic analysis  (Gibbs, 2012).  Focus groups. 6-12 participants/group. Semi-structured interview, open ended questions. | 27 nurses, 89% female, 72% > 30 years, average 5 years ED work experience.  Tertiary referral hospital in metropolitan Sydney, Australia. 60,000 hospital presentations annually. | Priority was given to assessments and treatment of acute illness, the medical side and the acute problems. Multiple patients were also considered as a barrier. This led to an inability to provide essential nursing care, e.g. lack of hydration, continence care and comfort measures. Basic nursing care was perceived long gone. This implied not meeting the nurses own care standards, in some cases resulting in patient humiliation. |
| Guedes dos Santos, J., Dias da Silva Lima, M., Pestana, A., Garlet, E., & Erdmann, A. (2013). Brazil. | To analyze the challenges for the management of care in a hospital emergency department. | Qualitative exploratory and descriptive study. | Content analysis technique was used, of a thematic analysis type.  Semi-structured interviews. | An intentional sample of 20 of the 32 nurses who worked in the section, had worked for more than six months in the emergency department.  University hospital located in the southern region of Brazil. | Patients that required attention that did not always correspond to the nursing team, according to the characteristics of the work unit and an excessive number of patients and inadequate physical space of emergency service rooms were main challenges. This could lead to care related to hygiene and comfort of patients being missed. Nurses were concerned about the quality of care provided in observation rooms of emergency service. |
| Innes, K., Elliot, D., Plummer, V., & Jackson, D. (2018). Australia. | To identify the activities and behaviors of waiting room nurses in emergency department settings. | Nonparticipant observational study. | Descriptive statistics and thematic analysis Braun and Clarke (2006).  Participant observation. | Nurses (n = 8) working in the WRN role. Emergency nursing experience of the participants varied from three years to greater than 15 years, with six participants having completed or currently undertaking postgraduate emergency nursing qualifications.  ED 1: Secondary referral, metropolitan hospital, major Australian city, 69289 ED presentations. ED 2: Tertiary referral, regional hospital, Victoria Australia, 53307 ED presentations. | The WRN contributed to patient safety in the waiting room by commencing episode of care in the waiting room, performing ongoing assessment and management of patients decreasing delays to care and detecting patient deterioration, involved patients and families in discussions and worked effectively with interprofessional teams to facilitate care. However, when the department was busy and overcrowded or when space was often used by medical officers, without consultation with the WRN could lead to assessments, interventions and reviews of patients being missed. |
| Enns, C., & Sawatzky, J-A. (2016). USA. | To gain insight into the meaning of caring from the perspective of emergency nurses. | Qualitative descriptive design | Content analysis.  Face-to-face interviews with individual participants. | A convenience sample of registered nurses (n = 17). The sample included emergency nurses and educators, age ranged from 26 to 61 years. Work experience ranged from 3 to 37 years.  Urban (n = 11) and rural (n = 6) facilities. | Heavy workload, lack of time, staffing issues, lack of management support, shift work, and lack of self-care were identified as factors affecting caring. These findings may be used to inform practice and improve the work milieu for emergency nurses. |
| Berben, S., Meijs, T., van Grunsven, P., Schoonhoven, L., & van Achterberg, T. (2012). The Netherlands. | To give insight into facilitators and barriers in pain management in trauma patients in the chain of emergency care (EMS and ED) in the Netherlands. | A qualitative approach. | Inductive thematic content analysis.  Individual interviews and focus groups meetings. | Six individual interviews with medical and nurse managers. Five focus group interviews were conducted with staff responsible for the actual pain management including paramedics, nurses, and physicians.  Two EMS ambulance services and three EDs from an academic trauma center, a teaching hospital and a regional general hospital. | This study identified five concepts as facilitators and barriers in pain management for trauma patients. Knowledge deficits, attitude problems and patient input were similar for the EMS and ED setting, despite the different positions, backgrounds, and educational levels of respondents. An overcrowded ED, high workload and the ED culture not primarily focused on patient comfort, was identified barriers. This could lead to systematic pain assessment and triage by MTS being omitted and a lack of follow-up in pain management. |
| Pavedahl, V., Holmström, I., Meranius, M., Schwarz, U., & Muntlin, Å. (2021). Sweden. | To explore how fundamental care needs of critically ill patients are being met in emergency rooms. | Descriptive exploratory design. | Ethnographic approach.  Non-participant observations inspired by Spradley. | A randomized sampling approach was used of RNs working in the emergency room. 50 RNs were eligible to participate and 23 (19 females and four males) were ultimately observed during the observation period.  One emergency room at an Swedish University hospital ED with approximately 54000 visits annually. | Even in a medical-technical environment, several fundamental care needs have been identified, whereby RNs work in an integrated manner to meet patients’ physical, psychosocial, and relational needs. It is crucial for RNs to work bedside to establish a patient–nurse relationship. Establishing such a relationship is also crucial for how and whether fundamental care needs are met. This study indicates that RNs are initially committed to and active in meeting both medical and nursing needs, but that this commitment decreases over time. |
| Kongsuwan, W., Matchim, Y., Nilmanat, K., Locsin, R., Tanioka, T., & Yasuhara, Y. (2016). Thailand. | To describe the meaning of nurses’ lived experiences in caring for critical and dying patients in ERs. | Hermeneutic phenomenological approach. | Hermeneutic phenomenological (van Manen’s, 1990).  Individual interviews. | Purposive/snowball sampling of twelve nurses working in the ER for at least two years with experience of caring for critical and dying patients in the ER.  The study was conducted in three ERs of tertiary hospitals. | This study offers an understanding of Thai nurses’ experiences in caring for critical and dying patients in the ERs. The nurses expressed that their ability to provide psychological support, prepare family members to accept the patient’s death were affected by not having time, the urgency of the context and the limited time available for care and the lack of competency and confidence. |
| Gorawara-Bhat, R., Wong, Alexandra., Dale, W., & Hogan, T. (2017). USA. | To understand ED nurses’ perceptions of assessing older patients’ pain and use emergent themes to guide optimal interventions for improving the quality of pain assessment in the ED. | Qualitative methodology. | In-depth, semi-structured interviews and participant observations. | A convenience sample representing the ED nurse population, (n = 20).  An academic urban adult ED in Midwest US. | Some of the challenges to assessing pain was; You can get backed up from just a very sick patient, busy with a critical patient, when a patient is more compromised, the hurried and crowded ED environment and when caring for a more critical patient at the time. This resulted in lack of adequate assessment/timing for reassessing. A persistent challenge for nurses involved making time for reassessment of a patient’s pain. |
| Lockett, J., Nelson, K., & Hales, C. (2021). New Zealand. | To explore the perspectives of New Zealand ED nurses on the provision of nursing care in ED during a pandemic, and to identify strategies they consider important for pandemic planning. | A descriptive exploratory qualitative design. | Inductive content and thematic analysis (Braun and Clarke’s six phases).  A semi-structured approach to interviews. | A convenience sample of 16 triage-trained registered nurses, experience in emergency nursing ranged from four to 20 years.  Two secondary and tertiary-level facilities EDs within the lower North Island of New Zealand. | New Zealand emergency nurses hold significant fears for how an influenza pandemic would impact on patients, and on their families and themselves. Several issues were raised across both EDs: Overcrowding, being time-poor, patients were often placed in ED corridors due to space constraints in overcrowded EDs and an imbalance in nurse-to-patient numbers was of particular concern. This in turn could lead to consequences for patients: The inability to provide all necessary patient care, and the provision of patient care in non-traditional treatment spaces, mainly ED corridors. Nurses expressed being unable to complete all nursing care tasks, treatment delays, patients being toileted in public, patients who have not had vital signs recordings for hours. Nurses expressed a fear that missing patients clinical deterioration and that patients safety was being compromised. |
| Eriksson, J., Gellerstedt, L., Hillerås, P., & Craftman Å. (2018). Sweden. | To explore registered nurses’ perceptions of safe practice in care for patients with an extended length of stay in the emergency department. | A qualitative descriptive methodology. | Qualitative content analysis with a latent approach as described (Krippendorff, 2013).  Semi-structured interviews. | Purposive sample of 14 RNs, eleven women and three men, with between 1–18 years of work experience as a RN in EDs. Two participants lacked experience of inpatient care.  Four hospitals in a conurbation in Sweden, three are university hospitals; two of them are connected university hospitals in Europe with 1600 beds. The third hospital has 85000 acute seeking patients; the fourth hospital is a central emergency hospital receiving 82000 visits in the ED. | Prolonged stays in the ED can lead to negative consequences for both patients’ safety and the RNs’ psychosocial experience. In addition, the RNs often find it impossible to perform safe practices of nursing and caring for patients with a prolonged ELOS in the ED. Some of the expressed reasons leading to perceived unsafe care was not knowing their patients well enough, being responsible for 20-25 patients, inability to follow routines, being overcrowded. Some of the nursing tasks being affected was a general lack of nursing, inability to take vital signs of patients. Further was documentation deemed unsatisfactory, risk assessment tools were not used, or patients’ vital signs were not noted. This had according to the nurses’ experiences lead to that patients had deteriorated in the ED and patient had sustained care related injuries. |
| Hitchcock, M., Gillespie, J., Crilly, J., & Chaboyer, W. (2014). Australia. | To explore and describe the triage process in the ED to identify potential problems and vulnerabilities that may impact the triage process. | - | Fieldwork methods. Thematic analysis (Braun & Clarke 2006).  Unstructured observer-only observation, field notes, informal and formal interviews. | Purposive sample of staff working in the ED setting that were involved in the triage process including triage nurses, emergency nurses, shift leaders, medical officers, ambulance officers and clerical staff.  One ED at a regional public teaching hospital in Queensland, Australia. The hospital had 450 beds. The ED operated on a 24-hour basis and had approximately 67000 patient presentations per year. | ED and hospital overcrowding, access block, patient flow blockages, ineffective communication, collabo- ration and teamwork and inexperience or a decreased level of competence of the triage nurse have all been identified as problems or potential vulnerabilities in the triage process. Extended time to triage, time to further assessment or re-assessment was observed resulting in missing important components of the patient’s condition, triage errors or inappropriate triage category allocation. |
| Avallin, T., Muntlin Athlin, Å., Björck, M., & Jangland, E. (2020). Sweden. | To explore, through the patient perspective, how patient–provider communication is linked to missed nursing care vs. meeting patients’ fundamental care needs. | A case study design. | Case study analysis.  Participant observation, informal interviews (Spradley, 1980). | A purposeful sample of 20 patients, 18 years or older with acute abdominal pain, six were accompanied by relatives.  One emergency department and two surgical wards at a Swedish university hospital. | This study lets us better understand the complexity behind missed nursing care in relation to communication failures and PCFC in relation to successful communications. The findings provide evidence that communication is decisive in meeting patients fundamental care needs and can help prevent the severe consequences of missed nursing care in an acute care setting. Little time or effort was required for providers to identify and meet individual care needs using communication, although this was often missed. |
| Hogan, K-A., Fothergill-Bourbonnais, F., Brajtman, S., Philips, S., & Wilson, K. (2016). Canada. | To describe the experience of emergency nurses who provide care for adult patients who die in the emergency department. | Interpretive descriptive approach. | Thematic analysis.  Individual audio-recorded interviews. | Six female and five male nurses. The mean age of participants and experience working in an emergency department was 37.4 years and 6.6 years.  Two emergency departments of a multisite university teaching hospital located in Ottawa, Ontario, Canada. Each of these emergency departments reports more than 60000 visits and more than 200 deaths annually. | The overriding message of this experience was that caring for adults who die in the emergency department is a difficult and challenging aspect of the nursing role. Factors that contribute to the difficulty are the busy environment, the lack of time to care for dying patients in an optimal manner, and the need to care for family members (often whom they had just met) who were experiencing a tragic moment in their lives. This could inhibit the nurses’ ability to provide compassionate care to dying patients, to establish relationships with patients and family members and to meet emotional and physical demands. |
| Warner, A., Saxton, A., Indig, D., Fahy, K., & Horvat, L. (2012). Australia. | To provide an understanding of women’s experiences in the ED when they present for a problem in early pregnancy and implications for clinical practice. | Qualitative study. | Thematic analysis approach.  In-depth semi-structured interviews. | 16 pregnant women under 20 weeks gestation and over the age of 16years.  Five EDs in the Hunter New England Area Health Service. The five hospitals selected comprised a tertiary referral hospital, three rural referral hospitals and a district health service in a rural and remote area. | Findings from this study suggest that ED staff play a vital role in the emotional care of women who suffer early pregnancy loss which has the potential to significantly help the mourning process. Some of the experiences were a lack of compassionate care, a lack of privacy, they would have liked more written information and some experienced significant distress. |
| Larivière-Bastien, D., deMontigny, F., & Verdon, C. (2019). Canada. | To identify characteristics of care management that may have contributed to the difficulties experienced by women presenting with miscarriage in the emergency department. | Phenomenological study. | Thematic data analysis (Paillé).  Semi- structured face-to-face interview. | 48 women ranging from 22 to 41 years old, who had experienced at least 1 miscarriage (20 weeks or less) in the past 4 years, had consulted 1 of the 4 emergency departments and been diagnosed with a miscarriage.  The 4 selected facilities represented a range of characteristics in terms of population served (urban, semi-urban, rural) and had a combined 147 ED beds. | Emergency nurses can improve emergency care for women experiencing miscarriage by listening to their concerns and providing accurate and sufficient information to reduce the physical and psychological effects on individuals, couples, and families. Participants reported a lack of information on the psychological aftermath of miscarriage, how much blood to expect and little or no information from nurses or physicians at discharge about post- miscarriage physical symptoms. All participants said the wait was distressing. |
| Wolf, L., Perhats, C., Delao,A., & Clark, P. (2017). USA. | To explore the effects of fatigue in emergency nurses on both cognition and work experience. | Constructivist approach. Qualitative exploratory design. | Inductive qualitative content analysis.  Focus-group, one-hour semi-structured interviewed. | 16 emergency nurses, 24->64 years old. 1-35 years’ experience as ED RNs.  Urban, suburban, rural Eds. 5001->100000 annual ED patient visits. 10 different US regions. | Fatigue as described by the sample of emergency nurses is an overwhelming mental and emotional exhaustion caused in part by overwork, extended shifts, and lack of breaks from the unpredictable demands typical of most ED settings. The high levels of fatigue reported by these emergency nurses resulted in frequent concerns about the quality and safety of patient care, including medical errors, delayed care, and failure to rescue. |
| Stein-Parbury, J., Gallagher, R., Fry, M., Chenoweth, L., & Gallagher, P. (2015). Australia. | To discover and disseminate information that will improve the patient/carer experience through enhanced practice delivery. | Qualitative design. | General interpretive methods  (Elo & Kyngäs, 2008).  Semi-structured interviews. | Ten patients 65–94 year, accompanied by a family carers, had at least one diagnosed chronic illness, living at home, presented to the ED with shortness of breath (n = 5) and chest pain (n = 5), with one older person also having fallen. Carers 52–91 years old were considered provided regular support to the person.  Referral university hospital in metropolitan Sydney, Australia. The ED of the hospital receives approximately 60000 presentations annually, one-third of which are people aged 65 years or older. | The ED experiences of older people and their carers reveal that their needs for information were not adequately met. Carers often advocated for the older person in order to gain information. They made efforts to have their needs met in a manner that was mindful that clinical staff in the ED were busy and overworked. Participants expressed that not enough nurses or not enough people (staff) was working leading to a lack of information among the participants. |
| Frank, C., Holmberg, M., Jernby, E. E., Hansen, A. S., & Bremer, A. (2022). Sweden. | To describe nurses’ experiences of dealing with older patients’ autonomy when cared for in Eds. | Qualitative study. | Lifeworld theory with a descriptive phenomenological approach.  Individual interviews. | Purposive sampling of 13 nurses. All participants were women (mean age 42 years. Their length of experience of working as a nurse in EDs varied from one to 38 years.  The study was conducted in two EDs in south Sweden. Approximately 67,000 adult patients were cared for at the two EDs in 2018. | Stressful work conditions, lack of time and medical things are important. A lack of organizational strategies for promoting older patients’ autonomy in EDs contributes to maintaining unjustified paternalistic care and questioning who has a legitimate responsibility for participating in care decisions in the ED. The nurses’ protection and promotion of older patients’ autonomy is dependent on the opportunity, ability and willingness to create a patient relationship where the patient’s voice and preferences are valued as important. |
| Pavedahl, V., Muntlin, Å., Summer Meranius, M., von Thiele Schwarz, U., & Holmström, I. K. (2022). Sweden. | To explore how RNs in the emergency room describe their work approach and prerequisites for meeting life-threateningly ill patients' care needs from the perspective of a person-centred fundamental care framework. | Descriptive design with a qualitative approach. | Thematic analysis according to Braun and Clarke (2020).  Individual interviews. | 14 RNs (11 females and three males) aged 28–61 years (mean 40.2). Mean working life experience in the emergency room ranged between 1 and 14 years (mean 6.1).  The study setting was an emergency room in an ED at a university hospital in Sweden with approximately 54,000 visits annually, of which more than 3,000 involved visits to the emergency room | An organizational focus on patient flow and the promotion of guidelines and checklists provide a structured approach for the initial care of patients, but are not adapted to provide them with person-centered care. The RNs' work approach in the emergency room, to meet patients' fundamental care needs, depends on personal and organizational prerequisites. According to the registered nurses new incoming patients were prioritized over helping existing patients and a constant in-flow and a prioritization of medical interventions contributed in failing to meet patients fundamental care needs. |
| Forsgärde, E. S., From Attebring, M., & Elmqvist, C. (2016). Sweden. | To disclose the meaning of patients and relatives lived experience of dis- satisfaction when visiting an emergency department. | Explorative study | Phenomenological hermeneutic method.  Interview. Open ended questions. | Two male patients age between 30 and 50 years and two male and two female relatives aged between 40 and 65 years.  The study took place at the ED of a central hospital in southern Sweden. the ED receives about 30,000 visitors each year. | Findings from this study showed that their meaning of dissatisfaction was similar: powerlessness, struggling for control, lacking knowledge and information, receiving and providing support. Patients and relatives felt abandoned during their wait time by not being seen or supported in their worries by health professionals. They felt disrespected when their accounts were received with skepticism or when they were given vague or misleading information. They suffered when being treated like objects during their ED visits. |
| Appel, G., Han, H., Re'Em, Y., Louka, C., Sundararajan, R., Tom, A., An, A., Difede, J., Avery, J., D; Zaidi, S R. (2024). United States | To produce a qualitative description of the impact of moral injury on medical providers during the COVID-19 pandemic | Convergent mixed-methods study design | Moral Injury Symptom Scale-HP (MISS-HP) survey and 60-minute interviews conducted via video. | 8 physicians and 6 nurses from a hospital in New York City, with most participants working in the emergency department (71%) and the rest in the medical intensive care unit (29%). | Average MISS-HP score was 49, with scores ranging from 29 to 73. No significant differences in scores between demographic groups.Interviews highlighted how omissions and commissions in professional duties created internal conflicts linked to feelings of guilt and blame. |
| Chiappinotto et al. (2023), Italy | To identify differences in the unfinished nursing care (UNC) occurrence, reasons, and consequences perceived by nurses caring for COVID-19 and non-COVID-19 patients during the pandemic | Systematic review | Data were collected from Medline, CINAHL, and Scopus databases using tools like the MISSCARE and UNC Survey. Data were compared across COVID-19 and non-COVID-19 patients during different waves of the pandemic | Hospital-based, five studies included, participants were nurses caring for COVID-19 and non-COVID-19 patients | UNC occurrence was higher among COVID-19 patients during the first wave and lower in later waves. Reasons included inadequate resources and work environment disruptions. No data on consequences were found. |
| Johnson, K D., Motavalli, M., Gray, D., Kuehn, C. (2014). United States | To identify the types and frequency of interruptions during the ED triage interview process. | Quality-improvement project | Focus group of emergency nurses to identify types of interruptions, validated through observations and tally sheets completed by triage nurses during 10 shifts (7 AM to 3 PM). | Single VA hospital emergency department in Cleveland, OH, with 12 acute care rooms and 6 “fast-track” rooms for non-acute patients, serving approximately 25,000 adult veteran patients annually. | On average, triage nurses were interrupted 48.2 times during an 8-hour shift (7 interruptions per hour). Only 22% of interruptions were related to patient care; most interruptions were not related to patient care, including opening the door (33%), providing conveniences to visitors (21%), and patients or family members asking “How much longer?” (14%).  Frequent interruptions can interfere with concentration, delay care, and affect the quality of care. |
| Morken, T., Alsaker, K., Johansen, I. H. (2016). England | To explore professional-patient interaction in aggressive incidents in emergency primary care. | Qualitative study with focus groups | Eight focus groups including a total of 37 nurses and physicians, aged 25–69 years, discussing their experiences of violence in emergency primary care. Discussions were recorded, transcribed, and analyzed using systematic text condensation. | 37 physicians and nurses with work experience from Norwegian emergency primary care centers. Mean age 41 years, mean work experience 9 years. Participants had work experience from various organizational and geographical subsets of emergency primary care clinics. | Identified three main themes regarding interaction in aggressive situations: unmet needs, involuntary assessment, and unsolicited touch.Aggression is more likely when patients' needs or personal boundaries are invalidated. Professional-patient interactions that include unmet needs, involuntary assessments, and unsolicited touch may trigger aggression. |
| Palese, A., Ambrosi, E., Fabris, F., Guarnier, A., Barelli, P., Zambiasi, P., Allegrini, E., Bazoli, L., Casson, P., Marin, M., Padovan, M., Picogna, M., Taddia, P., Salmaso, D., Chiari, P., Marognolli, O., Canzan, F., Saiani, L. (2016). England | To describe the natural history of emergency department-inserted peripheral venous cannula (PVC) site use, the occurrence and severity of PVC-related phlebitis, and associations with patient, PVC, and nursing care factors. | Prospective explanatory pragmatic study | The Visual Infusion Phlebitis Scale (VIPS) and the MISSCARE Survey  Daily observation of the first PVC inserted in emergency departments until removal. Data on patient, PVC, nursing care, and organizational variables were collected. Phlebitis was assessed using VIPS.The amount of care missed during the last shift was measured using the MISSCARE survey. | 1262 patients treated as urgent cases in emergency departments.  12 medical units in 12 hospitals located in northern Italy. | The prevalence of PVC-related phlebitis was 31%. Cumulative incidence was almost 20% three days after insertion and over 50% five days after insertion. Being in a specialized hospital and receiving more nursing care were protective against PVC-related phlebitis.  The risk of phlebitis was reduced by 1% for each additional minute of daily nursing care. |
| Zhang, X., Zhou, J., Chen, F., Yang, J., Jiang, Z., De Jesus, D. H. (2024). England | To examine the current situation and relationship between missed nursing care (MNC) and job satisfaction among frontline nurses in a hospital dedicated to treating COVID-19 patients in China. | Cross-sectional study | Convenience and snowball sampling techniques were used to recruit frontline nurses working in a hospital for treating COVID-19 patients from November to December 2022. The questionnaires included sociodemographic information, job satisfaction, and the MISSCARE survey. | 304 frontline nurses deployed to Jiangjunshan Hospital in Guizhou Province, China, to care for COVID-19 patients. Participants had to have worked in the hospital for at least one month, provided nursing care directly to COVID-19 patients, and volunteered to participate. | Frontline nurses' job satisfaction was high, and their MNC was low. The highest MNC was "offer rehabilitation care and guidance to patients in need every day."  The most reported reasons for MNC were "urgent patient situations." There were statistically significant differences in job satisfaction and MNC scores among participants' demographic variables. A negative correlation was identified between frontline nurses' job satisfaction and MNC. Higher job satisfaction was associated with lower levels of MNC. |
